# New criteria for gestational diabetes in Tehran, Iran

**Published:** 2012-05

**Authors:** Samira Behboudi Gandevani, Ahia Garshasbi, Sara Shahpari Niri, Mohammad Mehdi Naghizade

**Affiliations:** 1 *Department of Reproductive Health, Tarbiat Modares University, Tehran, Iran.*; 2 *Department of Obstetrics and Gynecology, Shahed University, Tehran, Iran.*; 3 *Department of Biostatistics, Tarbiat Modares University, Tehran, Iran.*

**Keywords:** *Carpenter and Coustan criteria*, *Gestational diabetes mellitus*, *Iranian pregnant women*, *NDDG criteria*, *New diagnostic criteria*

## Abstract

**Background:** Gestational diabetes mellitus (GDM) is common problem during pregnancy. Diagnostic criteria of this problem are based on foreign population. Because of differences in racial, cultural, and nutritional characteristics, we need to determine these criteria are suitable for Iranian population.

**Objective:** To determine whether different diagnostic criteria of gestational diabetes mellitus (GDM) are suitable for Iranian population.

**Materials and Methods:** Prospective study was performed on 617 pregnant women. 1804 subjects referred for 50 g glucose challenge test (GCT) between 24th and 28^th^ weeks of gestation. 617 women with abnormal GCT (blood glucose ≥130 mg/dl) underwent 100-g 3-h oral glucose tolerance test (OGTT). The results were classified by three diagnostic criteria: new “Iranian” diagnostic criteria based on the results from the 100-g 3-h OGTT performed in healthy participating women; the Carpenter and Coustan (CC) criteria; and the National Diabetes Data Group (NDDG) criteria. Obstetric and neonatal outcomes were recorded.

**Results:** With 89% as the statistical cutoff value for the 100-g 3-h OGTT, the new diagnostic criteria were 92, 179, 153, and 121 mg/dL at 0, 60, 120, and 180 min. The K value was 0.945 for the new criteria vs. the CC criteria and 0.657 for the new criteria vs. the NDDG criteria (p<0.001). In women with GDM, the incidence rates of adverse outcomes by the new and CC criteria were similar, but higher than NDDG criteria (p<0.05).

**Conclusion:** Carpenter and Coustan criteria are applicable to Iranian pregnant women for diagnosis of GDM.

## Introduction

Gestational diabetes mellitus (GDM), defined as diabetes first discovered or with onset during pregnancy, particularly in the second trimester, is associated with increased risk of several adverse infant and maternal outcomes (1, 2). Clinical recognition of GDM is important because it may lead to appropriate perinatal management. Results from a randomized controlled trial show that treatment of GDM by means of dietary advice, blood glucose monitoring, and insulin therapy, if required, reduces the rate of serious perinatal complications (3, 4) and promote postpartum diabetes-prevention strategies (5-9). 

The criteria for abnormal glucose tolerance in pregnancy are based on oral glucose tolerance test (OGTT) (10-12). Because of differences in racial, cultural, and nutritional characteristics, we designed this study to determine whether foreign different diagnostic criteria for the diagnosis of gestational diabetes mellitus (GDM) are suitable for Iranian pregnant women and introduce the new Iranian criteria for diagnosis of gestational diabetes.

## Materials and methods

Prospective study for diagnosing of GDM was performed on 617 pregnant women. This study was approved by the Ethical Committees of Shahed University Tehran, Iran. The participants were drawn from two prenatal clinics in Tehran, after obtaining informed consent for the scientific use of the data. Women who had glucose intolerance before pregnancy or had history of GDM in previous pregnancies with per persistent abnormal or undetermined glucose tolerance were not included in the study.

At first, the 1804 pregnant women were referred for a 50 g oral glucose challenge test (GCT), for screening of GDM, between 24^th ^and 28^th^ weeks of gestation. GCT was performed regardless to the time of last meal. One hour after 50 g glucose consumption, plasma glucose concentration (glucose oxidase method) was measured. 

However, when risk factors such as positive family history of diabetes, age >25 years, pre-pregnancy overweight, personal history of GDM, glucosuria and history of macrosomia were present, GCT was performed at the 14^th^-18^th^ weeks of gestation. In the latter group, when the GCT result was negative, a further GCT was performed at 24^th^-28^th^ weeks of gestation. 

In total, 617 subjects had abnormal GCT (blood glucose level ≥130 mg/dL). These women were divided into two groups; the group who did not have any risk factor for GDM (247 women), and the high-risk pregnancy group which had at least one high-risk factor for GDM (370 women). The risk factors for GDM were defined as follows: hypertension; blood pressure ≥140/ 90mm Hg, hyperlipidemia; a serum triglyceride level of 300 to 400 mg/dL and a high-density lipoprotein cholesterol level of 30 mg/dL or less), family history of diabetes mellitus; at least one of the pregnant women's parent, brothers, sisters, aunts, uncles, or grandparents had diabetes, personal history of gestational diabetes; according to the criteria issued by NDDG (10), poor history of previous obstetrical outcomes; 2 or more spontaneous abortion, prior fetal malformation, prior fetal death, and prior stillbirth (prior fetal death was defined as an unexplained death in uterus at gestational age of 28 weeks or later), previous macrosomia in offspring; birth weight ≥4000g, oligohydramnios amniotic volume ≤300 mL), glucosuria during current pregnancy and obesity (was defined as the women's mean body mass index, calculated as weight in kilograms divided by the square of height in meters, was lower than 27, were the normal pregnancy group. All of women with abnormal GCT underwent an oral glucose tolerance test (OGTT 100-g 3-h) within 1 week after the abnormal screening test to determine whether or not they had gestational diabetes mellitus (9, 13) and were classified according to three different sets of diagnostic criteria: (1) new "Iranian" diagnostic criteria, based on data obtained from the 100-g 3-h OGTT performed in the healthy participants; (2) the Carpenter and Coustan criteria (5) (the references values obtained at 0, 60, 120, and 180 min were 95, 180, 155 and 140 mg/dL); and (3) the NDDG criteria (14) (the reference values at 0, 60, 120, and 180 min were 105, 190, 165 and 145 mg/dL). 

When all values were less than the reference value, the pregnant woman was considered to have a normal pregnancy; when only one value was equal to or greater than the reference values she was considered to have gestational-impaired glucose tolerance (GIGT); and when two or more values were equal to or greater than the reference values she was considered to have gestational diabetes (GDM). Obstetric outcomes were recorded. These outcomes included cesarean delivery, preterm births (gestational age at birth <37), low birth weight (neonatal weight <2500g), macrosomia (neonatal weight ≥4000g), stillbirth and fetal distress (low Agpar score <7).

**Statistical analysis**

All statistical analyses were performed using SPSS version 11.5 for Windows (SPSS Inc., Chicago, IL). Skewness and Kurtosis were used to detect the distribution of a variable. When a variable was distributed in a parametric manner, the results were presented as mean±SD. Comparisons between groups were performed using the unpaired t-test. To compare proportions (qualitative variables) the chi-square test and the Fisher exact test (when expected values were <5%) were used. Statistical significance was set at the 95% level (p<0.05).

## Results

Table I show the distribution of venous plasma glucose level obtained with the 100-g 3-h OGTT in women's with an abnormal GCT (blood glucose level ≥130 mg/dl). The test results were approximately normally distributed for the 247 healthy pregnant women. According to different skew and kurtosis distribution, we chose 89% as the statistical cutoff value for the 100-g 3-h OGTT. 

The values of new diagnostic criteria assessed in this study, were 92, 179, 153 and 121 mg/dL at 0, 60, 120, and 180 min. According three sets of criteria, there were more women with GDM or impaired glucose tolerance in the high risk group than in the low risk-pregnancy group, but the differences weren’t significant (p>0.05) (Table II). It may be resulted from the small number of samples.

Table III and IV indicate that definitions of GDM by the three set of criteria. Kappa value was 0.945 for the new criteria vs. the Carpenter and Coustan criteria and 0.657 for the new criteria vs. the NDDG criteria (p<0.001). A consistency check showed more consistency between the new criteria and the Carpenter and Coustan criteria. The prevalence of GDM was 7.7% by the new criteria, 7.2% by Carpenter and Coustan criteria, and 4.1% by the NDDG criteria.

All women found to have GDM by NDDG criteria, were treated with a strict diabetic protocol. Dietary recommendations were given to maintain their plasma glucose levels during fasting at less than 104 mg/dl and 2 h postprandial levels at less than 120 mg/dl. When diet treatment could not achieve this goal, then insulin therapy was initiated (12, 15). Overall 10 patients needed insulin treatment. Obstetrics and neonatal outcomes are given in table V. Mean±SD gestational age at birth was 38.04±2.46 weeks and neonatal weight was 3243.4±410.3 g.

**Table I:** Distribution of Venous Plasma Glucose (mg/dL).

		**x±s**	**89 percentile**	**Kurtosis**	**Skew**
Low-risk -pregnancy woman (n=247)					
	Fasting	82±9.32	92	1.29±0.416	0.88±0.209
	1h	162.62±21.11	179.26	3.06±0.417	0.06±0.211
	2h	126.67±23.75	153	2.41±0.417	0.66±0.211
	3h	94.45±18.79	121	0.11±0.417	0.64±0.211
High-risk pregnancy woman (n=370)					
	Fasting	86.01±12.09	100	10.20±0.221	1.89±0.111
	1h	174.20±29.59	206	4.94±0.221	1.59±0.111
	2h	135.68±32.475	171	2.92±0.221	1.205±0.111
	3h	100.44±26.52	133	0.86±0.221	0.86±0.111

**Table II:** Number of women with GDM and GIGT in the high risk group and low risk group

		**GDM**	**GIGT**	**p-value**
High risk group (n=370)				
	New criteria	73	84	0.62
	C&C criteria	67	78	
	NDDG criteria	40	60	
Low risk group (n=247)				
	New criteria	66	75	0.71
	C&C criteria	64	71	
	NDDG criteria	34	51	

GDM: Gestational Diabetes Mellitus.

GIGT: Gestational-Impaired Glucose Tolerance.

**Table III:** The constitution of GDM and GIGT by new criteria and Carpenter and Coustan criteria (n=617).

		**Carpenter and Coustan criteria**			**Total**
		**GDM**	**GIGT**	**Normal**	
New criteria					
	GDM (n)	131	7	1	139
	GIGT (n)	0	142	17	159
	Normal (n)	0	0	319	319
Total (n)		131	149	337	617

GDM: Gestational Diabetes Mellitus.

GIGT: Gestational-Impaired Glucose Tolerance.

**Table IV:** The constitution of GDM and GIGT by new criteria and NDDG criteria (n=617).

	**NDDG criteria**			**Total**
	**GDM**	**GIGT**	**Normal**	
**New criteria**				
GDM (n)	74	27	38	139
GIGT (n)	0	84	74	158
Normal (n)	0	0	320	320
Total (n)	74	111	432	617

GDM: Gestational Diabetes Mellitus.

GIGT: Gestational-Impaired Glucose Tolerance.

NDDG: National Diabetes Data Group.

**Table V:** Obstetric and neonatal outcomes of GDM and GIGT by three sets of diagnostic criteria

		**High risk group (n=370)**			**Low risk group (n=247)**		
		**New criteria**	**C&C criteria**	**NDDG criteria**	**New criteria**	**C&C criteria**	**NDDG criteria**
Cesarean delivery (n=393)							
	GDM	69 (17.5%)	64 (16.2%)	35 (8.9%)	63 (16%)	61 (15.5%)	29 (7.3%)
	GIGT	75 (19%)	73 (18.5%)	53 (13.4%)	68 (17.3%)	64 (16.2%)	46 (11.7%)
Preterm birth (n=31)							
	GDM	8 (25.8%)	7 (22.5%)	7 (12.2%)	6 (19.3%)	6 (19.3%)	3 (9.6%)
	GIGT	10 (32.2%)	10 (32.2%)	10 (32.2%)	7 (22.5%)	7 (22.5%)	5 (16.1%)
Low birth weight (n=37)							
	GDM	12 (32.4%)	10 (27%)	4 (10.8%)	10 (27%)	9 (24.3%)	3 (8.1%)
	GIGT	10 (27%)	11 (29.7%)	11 (29.7%)	11 (29.7%)	11 (29.7%)	8 (21.6%)
Macrosomia (n=31)							
	GDM	10 (32.2%)	9 (29%)	4 (12.9%)	8 (25.8%)	7 (22.5%)	4 (12.9%)
	GIGT	3 (9.6%)	2 (6.4%)	7 (22.5%)	2 (6.4%)	5 (16.1%)	6 (19.3%)
Fetal distress (n=29)							
	GDM	10 (34.4%)	8 (27.5%)	3 (10.3%)	7 (24.1%)	7 (24.1%)	2 (6.8%)
	GIGT	5 (17.2%)	5 (17.2%)	10 (34.4%)	5 (17.2%)	4 (13.7%)	5 (17.2%)
Stillbirth (n=3)							
	GDM	2 (66.6%)	2 (66.6%)	1 (33.3%)	2 (66.6%)	1 (33.3%)	1 (33.3%)
	GIGT	1 (33.3%)	1 (33.3%)	2 (66.6%)	0 (0%)	0 (0%)	0 (0%)

C & c criteria: Carpenter and Coustan criteria.

NDDG: National Diabetes Data Group criteria.

## Discussion

GDM is defined as glucose intolerance with onset or first time recognition during pregnancy. Insulin or only diet therapy is used for treatment (16). 

According to World Health Organization classification, Gestational diabetes mellitus is a grade A disease. There are different criteria for diagnosing approach of gestational diabetes. The World Health Organization (17) recommends the 75-g 2-h OGTT approach, which is often used in Europe. In 1964, O’Sullivan and Mahan (18) used 100-g, 3-h oral glucose tolerance test (OGTT) to diagnose GDM. In this study 752 women underwent a 100-g, 3-h OGTT during the second or third trimester of pregnancy using the Somogyi- Nelson technique as a chemical method. 

Following years most laboratories used analyzing blood glucose levels with this threshold and physicians accepted these thresholds. In 1979, the National Diabetes Data Group (NDDG) (19) suggested adjusting thresholds. However, in 1982, Carpenter and Coustan (11) recommended a new enzymatic method to measure plasma glucose levels. 

These changes resulted in lower diagnostic plasma glucose thresholds compared with the NDDG thresholds. Both the NDDG and the Carpenter and Coustan diagnostic criteria have been used by practitioners, and no specific recommendations regarding GDM diagnostic criteria were provided by the Fifth International Workshop Conference on Gestational Diabetes Mellitus (13). In American Diabetes Association recommendation 2010, the one approach is perform initial screening by measuring plasma or serum glucose 1 h after a 50-g load of ≥140 mg /dl identifies ~80% of women with GDM, while the sensitivity is further increased to ~90% by a threshold of ≥130 mg/dl (20). The new standards set by ADA recommendation in 2011 detection and diagnosis of gestational diabetes mellitus has been revised to reflect use of the 75-g oral glucose tolerance test. 

It is recommended that; 1) universal screening at 24-28 weeks of gestation (2010 ADA standards recommended selective screening based on risk factors) and 2) an oral glucose tolerance test with a diagnostic fasting plasma glucose of ≥92 mg/dL (4.5mmol/L) (much lower than the WHO criteria of ≥126 mg/dL [7.0 mmol/L] commonly used in clinical practice in Europe). Furthermore, diabetes is diagnosed when only one abnormal value is detected (whereas in the 2010 standards two abnormal values were needed) (21). However, Iran is an Asian population. There is highest prevalence of diabetes mellitus in Asia (22). Maybe it depends on differences in racial, cultural, and nutritional characteristics. In addition, domestic hospitals in Iran adopted different foreign criteria and there is no evidence to show us which one is appreciate for Iranian population. 

The aim of this study was to explore which criteria are suitable for Iranian pregnant women. In this study we showed that the Iranian criteria for plasma glucose level were lower than the WHO criteria and NDDG criteria but similar to the criteria recommended by Carpenter and Coustan criteria. Likewise in fasting threshold is similar to ADA recommendation 2011 (20). Some other Asian population adopted these criteria too (23, 24). The prevalence of GDM increased by 56% (from 7.2%, 131 out of 1804 pregnant women, to 4.1%, 74 out of 1804 pregnant women), when we use the glucose thresholds modified by Carpenter-Coustan instead of the glucose thresholds modified by the NDDG. 

The adverse outcomes of GDM such as Cesarean delivery, macrosomia, preterm birth, low birth weight, fetal distress, and stillbirth were similarly predicted with the three sets of criteria. In women with GDM, the incidence rates of preterm birth, low birth weight, fetal distress and macrosomia by the new criteria and the Carpenter and Coustan criteria were similar, but higher than the rates calculated with the NDDG criteria (p=0.001). The treatment received by the subjects who had GDM with NDDG criteria is one of the possible reasons for such a finding.

The incidence rate of adverse outcome of GDM in high-risk group were higher than low risk group but there aren’t any significant differences between them (p=0.08). In Iran there are limited resources, obviously, the decision to use the Carpenter-Coustan thresholds will result in higher prenatal care costs to monitor and treat the additional women diagnosed with GDM. But some literatures suggest that women with untreated GDM by Carpenter-Coustan plasma glucose thresholds who did not meet the NDDG criteria had higher rates of costly adverse outcomes and perinatal complications than normoglycemic women (25).

However, Identification of GDM may lead to more effective strategies like healthy nutrition and physical activity for primary prevention of diabetes in these populations (26, 27). It is not known whether the cost of these interventions will be outweighed by the money saved by preventing perinatal complications among women with the lower Carpenter- Coustan thresholds. But it seems that the decision to use the Carpenter-Coustan thresholds could save the money (1). 

In summary, the diagnostic thresholds of GDM used in this study were similar to those of Carpenter and Coustan, which suggested that the Carpenter and Coustan are suitable for Iranian women.
